# Impacts on Postoperative Bleeding of Surgery for Head and Neck Malignancies While Continuing Antithrombotic Agents

**DOI:** 10.1055/s-0044-1791644

**Published:** 2025-01-29

**Authors:** Koji Ushiro, Ryo Asato, Hiroki Ishida, Chisato Chikugo, Yukiko Ito, Takuya Tsuji, Jun Tsuji

**Affiliations:** 1 Department of Otolaryngology, Head and Neck Surgery, National Hospital Organization Kyoto Medical Center, Kyoto, Japan

**Keywords:** head and neck surgery, antithrombotic agent, postoperative bleeding, postoperative complication

## Abstract

**Introduction** Perioperative management of antithrombotic agents may affect bleeding and lead to thromboembolic complications, but there is no consensus on optimal protocol in head and neck surgery.

**Objective** To explore the effect of antithrombotic agents on postoperative bleeding.

**Methods** We compared clinical characteristics, type of surgery, antithrombotic agents, continued use of medication or not, and frequency of postoperative bleeding among patients who were receiving antithrombotic therapy at the time of their decision to undergo surgery for head and neck malignancies, from 2008 to 2022.

**Results** A total of 168 patients were included. There was no significant difference in the incidence of intraoperative blood loss or postoperative bleeding between the group that underwent surgery while on antithrombotic therapy and those that underwent surgery after antithrombotic therapy was discontinued. In particular, there was no increase in bleeding complications with antiplatelet agents, regardless of the type or number of agents used.

**Conclusion** Surgery for head and neck malignancies with continued antiplatelet therapy may not increase bleeding complications, regardless of the type of antiplatelet therapy and even when multiple agents are taken.

## Introduction

The number of patients receiving antithrombotic therapy for cardiovascular disease is increasing in the aging Japanese population. Because patients with head and neck malignancies are relatively older, it appears that surgery is more frequent among patients taking antithrombotic agents. Continuing this drug therapy may increase risk for perioperative bleeding complications, whereas discontinuing it may increase risk for thromboembolic events.

With the exception of a few limited areas such as dental extractions, 1 2 ophthalmic surgery, 3 4 and gastrointestinal endoscopic procedures, 5 6 there is still no consensus on the optimal protocol for the perioperative antithrombotic management. There are also no guidelines for the use of antithrombotic agents in the perioperative period of head and neck surgery.

Surgery still plays a major role in the curative treatment of head and neck malignant tumors, and it is desirable that the use of antithrombotic agents have a minimal impact on the decision of treatment strategy. However, the head and neck region require careful consideration on whether or not to continue antithrombotic agents in the perioperative period because postoperative bleeding in head and neck surgery could lead to fatal adverse events such as airway stenosis due to its compression and venous congestion.

Since 2015, the study's center has followed a policy of continuing antithrombotic agents for any type of surgery. Therefore, in this study, we retrospectively examined the effects of this treatment plan to analyze the relationship between the continuation of different antithrombotic agents in the perioperative period and postoperative bleeding.

## Methods

A retrospective chart review was performed on patients with head and neck malignancies who underwent surgical treatment with curative intent at the Department of Otolaryngology – Head and Neck Surgery, National Hospital Organization Kyoto Medical Center between January 2008 and August 2022. All patients were receiving antithrombotic therapy at the time of the decision to undergo surgery. Patients with recurrence after initial treatment were only included, if surgery was performed with curative intent. Those who underwent surgery for diagnostic purposes and who were not pathologically diagnosed as malignant were excluded.

Data collection included patients' preoperative characteristics, surgery type, operative time, intraoperative blood loss, and postoperative complications like bleeding and thromboembolic events. These postoperative bleeding events were defined as surgical site bleeding above the usual level, as assessed by the surgeon. Major postoperative bleeding events were those requiring surgery or procedures for hemostasis.

Since there was a wide variety of diseases to be studied and various surgeries were performed with curative intent, the surgical procedures were classified according to whether they included the following characteristics: transnasal or trasnsoral without cervical skin incision; with concurrent neck dissection; contaminated; with maxillary or mandibular bone resection, including concurrent tracheostomy; and with concurrent reconstruction. Although energy devices have been used in head and neck surgery since about 2011, our institution has continued to follow a policy of not using energy devices except for surgery with concurrent reconstructive surgery.

Detailed data on the perioperative antithrombotic therapies were collected, including the type of oral agents used before surgery, and management at the time of the surgery, including continuation, discontinuation, and alternative treatments such as heparin bridging therapy.

If antithrombotic agents were to be continued, they were taken orally until the morning of surgery and resumed on the day after. If oral intake was not possible postsurgery, the drug was administered via a feeding tube. On the other hand, if antithrombotic agents were discontinued, it happened various days before and resumed 2 to 3 days after surgery.

Oral antithrombotic agents included antiplatelet and anticoagulant agents. Oral antiplatelet agents before the surgery included aspirin, thienopyridines (clopidogrel, ticlopidine, or prasugrel), cilostazol, and other antiplatelet agents (dipyridamole, limaprost alfadex, sarpogrelate, and beraprost). Continuation or discontinuation of the perioperative management was limited to the major antiplatelet agents – aspirin, thienopyridines, or cilostazol – regardless of the use of other agents. Oral anticoagulants included vitamin K antagonists (VKA) and direct oral anticoagulants (DOAC). Intravenous injection of unfractionated heparin (UFH) was used in all patients undergoing bridging.

Each patient receiving two antithrombotic agents was assigned to the continuation group if either medication was maintained, and to the discontinuation group if both were not. If both antiplatelet and anticoagulant agents were administered, the continuation or discontinuation of each agent was recorded.

Statistical analysis was performed using R software (R foundation for Statistical Computing, Vienna, Austria), version 4.2.2. We described the clinical characteristics using median and interquartile range (IQR) for continuous variables and number and percentage (%) for categorical variables. We fit unadjusted and adjusted logistic regression models using generalized estimating equations to examine associations between the clinical characteristics and postoperative bleeding. All *p* -values < 0.05 were statistically significant.

This study was approved by the institutional review board at National Hospital Organization Kyoto Medical Center.

## Results

The present study included a total of 168 patients who received preoperative antithrombotic therapy and underwent surgical treatment with curative intent for head and neck malignancies. The primary sites were oral cavity in 58 cases, sinonasal in 5, oropharynx in 20, hypopharynx in 13, larynx in 19, cervical esophagus in 2, salivary gland in 6, thyroid gland in 37, external auditory canal in 1, and unknown primary in 7 cases.

We divided patients into two groups, those who continued antithrombotic therapy at the time of surgery (continuation group) and those who discontinued antithrombotic therapy (discontinuation group), and compared the clinical characteristics, types of surgery, and outcomes between the two groups. The clinical characteristics of patients in each of the continuation and discontinuation groups are shown in Table 1 . There were no differences in terms of age, gender, and performance status between the two groups, but, in the continuation group, there was a slightly higher number of patients with an American Society of Anesthesiologists' (ASA) physical status classification ≥ 3. There were no differences in operative time or types of surgery between the two groups.

**Table 1:** Clinical characteristics and type of surgery among the study sample

Characteristic	Discontinuation group (N = 63) ^a^	Continuation group (N = 105) ^a^	*p* -value ^b^
Age (year)	74.0 (67.0–80.0)	74.0 (70.0–81.0)	0.352
Gender			0.856
* Male*	47 (74.6%)	77 (73.3%)	
* Female*	16 (25.4%)	28 (26.7%)	
Performance status > 2	13 (20.6%)	31 (29.5%)	0.205
ASA score > 3	29 (46.0%)	63 (60.0%)	0.078
Recurrent disease	13 (20.6%)	20 (19.0%)	0.802
Prior radiotherapy	8 (12.7%)	6 (5.7%)	0.113
Operative time (minutes)	217.0 (119.5–302.5)	172.0 (95.0–273.0)	0.260
Transnasal or transoral surgery only	13 (20.6%)	21 (20.0%)	0.921
Neck dissection	41 (65.1%)	58 (55.2%)	0.209
Contaminated	17 (27.0%)	25 (23.8%)	0.645
Bone resection	12 (19.0%)	19 (18.1%)	0.878
Tracheostomy	20 (31.7%)	33 (31.4%)	0.966
Reconstruction	12 (19.0%)	22 (21.0%)	0.766
Local flap	8 (12.7%)	5 (4.8%)	0.077
Free flap	7 (11.1%)	17 (16.2%)	0.362

**Abbreviation:** ASA, American Society of Anesthesiologists.

**Notes** : ^a^ Median (interquartile range) or n (%). ^b^ Wilcoxon rank-sum, Pearson Chi-squared, and Fisher exact tests.

The number of patients taking different types of antiplatelet and anticoagulant agents for each discontinuation and continuation group is shown in Table 2 . As for antiplatelet agents, 105 patients were taking aspirin, and 66 of them (62.9%) underwent surgery while continuing. Additionally, 42 patients were taking thienopyridines and 14 patients were taking cilostazol, and of these, 24 (57.1%) and 8 (57.1%) underwent surgery while continuing. As for anticoagulant agents, 15 patients were taking VKA and 13 DOACs, of whom, 5 (33.3%) and 12 (92.3%) underwent surgery while continuing medicine intake.

**Table 2:** Type of antithrombotic agent used among the study sample

Antithrombotic agents	Discontinuation group	Continuation group
*Antiplatelet agents*		
Aspirin	39	66
Thienopyridines	18	24
Cilostazol	6	8
Others	6	3
*Anticoagulant agents*		
Vitamin K antagonists	10	5
Direct oral anticoagulants	1	12
Combination therapy		
Aspirin + thienopyridines	5	10*
Aspirin + cilostazol	3	1
Thienopyridines + cilostazol	0	2
Antiplatelet agents + vitamin K antagonists	2	1

**Notes:** Thienopyridines include clopidogrel, ticlopidine, or prasugrel. Other antiplatelet agents include dipyridamole, limaprost alfadex, sarpogrelate, and beraprost. *Seven patients continued on both agents and three, on aspirin alone. ^†^ One patient continued on both agents and another, on cilostazol alone.

Furthermore, a total of 24 patients were taking a combination of antithrombotic agents. Dual antiplatelet therapy (DAPT) is a combination treatment of aspirin and thienopyridines for a certain period of time after endovascular procedures and was performed in 15 cases. Of these, 8 (53.3%) underwent surgery while continuing both agents, and 2 (13.3%) underwent surgery while continuing aspirin only. There were 7 of the 63 patients who discontinued antiplatelet agents preoperatively, and 2 of the 10 patients who discontinued VKA preoperatively received intravenous UFH.

The outcomes of patients both the continuation and discontinuation groups are shown in Table 3 . There was no difference in operative time, intraoperative blood loss, wound complications, systemic complications, or incidence of overall and major postoperative bleeding between the two groups. Additionally, there was no difference in any of the outcomes between the groups when analyzed separately for antiplatelet and anticoagulants agents ( Tables 4 5 ). None of the 7 patients receiving intravenous UFH had postoperative overall or major bleeding. Among the antiplatelet agents, thienopyridines with potent antiplatelet effects were used preoperatively in 42 patients, of which 24 (57.1%) were in the continuation group, but overall postoperative bleeding occurred in only one case in the discontinuation group, and no major postoperative bleeding was observed in either group. Additionally, regarding the 15 patients receiving DAPT, the overall postoperative bleeding was observed in 1 of 7 patients in the discontinuation group. No major postoperative bleeding was observed in either group.

**Table 3:** Patient outcomes

Characteristic	Discontinuation group (N = 63) ^a^	Continuation group (N = 105) ^a^	*p* -value ^b^
Operative time (minutes)	217.0 (119.5–302.5)	172.0 (95.0–273.0)	0.260
Intraoperative blood loss (mL)	140.0 (30.0–375.0)	70.0 (15.0–220.0)	0.059
Wound complication	13 (20.6%)	10 (9.5%)	0.043*
Systemic complication	5 (7.9%)	5 (4.8%)	0.504
Thromboembolic complication	1 (1.6%)	1 (1.0%)	> 0.999
Overall postoperative bleeding	8 (12.7%)	11 (10.5%)	0.660
Major postoperative bleeding	5 (7.9%)	7 (6.7%)	0.765

**Notes:**^a^ Median (interquartile range) or n (%). ^b^ Wilcoxon rank-sum, Pearson Chi-squared, and Fisher exact tests. * *p* < 0.05.

**Table 4:** Patient outcomes regarding the use of antiplatelet agents

Characteristic	Discontinuation group (N = 55) ^a^	Continuation group (N = 88) ^a^	*p* -value ^b^
Operative time (minutes)	223.0 (125.0–313.0)	181.5 (104.0–328.0)	0.359
Intraoperative blood loss (mL)	150.0 (30.0–390.0)	77.5 (20.0–300.0)	0.195
Wound complication	11 (20.0%)	8 (9.1%)	0.062
Systemic complication	5 (9.1%)	5 (5.7%)	0.508
Thromboembolic complication	1 (1.8%)	1 (1.1%)	> 0.999
Overall postoperative bleeding	7 (12.7%)	7 (8.0%)	0.350
Major postoperative bleeding	5 (9.1%)	4 (4.5%)	0.306

**Notes:**^a^ Median (interquartile range) or n (%). ^b^ Wilcoxon rank-sum, Pearson Chi-squared, and Fisher exact tests.

**Table 5:** Patient outcomes regarding the use of anticoagulant agents

Characteristic	Discontinuation group (N = 10) ^a^	Continuation group (N = 18) ^a^	*p* -value ^b^
Operative time (minutes)	171.0 (80.0–180.3)	141.0 (95.5–182.3)	0.867
Intraoperative blood loss (mL)	90.0 (15.0–135.0)	77.5 (1.3–77.5)	0.170
Wound complication	2 (20.0%)	2 (11.1%)	0.601
Systemic complication	0	0	
Thromboembolic complication	0	0	
Overall postoperative bleeding	2 (20.0%)	4 (22.2%)	> 0.999
Major postoperative bleeding	1 (10.0%)	3 (16.7%)	> 0.999

**Notes:**^a^ Median (interquartile range) or n (%). ^b^ Wilcoxon rank-sum, Pearson Chi-squared, and Fisher exact tests.

The association of continuing antithrombotic agents at the time of surgery with overall and major postoperative bleeding is shown in Table 6 . Continuation of antiplatelet agents wasn't associated with either overall or major postoperative bleeding, but continuation of anticoagulant agents was associated with higher odds of both overall and major postoperative bleeding, although the difference was not significant.

**Table 6:** Associations involving the continuation of the antithrombotic agents at the time of surgery with overall and major postoperative bleeding

Characteristic (chosen therapy)	Overall bleeding (unadjusted OR)	95% CI	*p* -value	Major postoperative bleeding (unadjusted OR)	95% CI	*p* -value
Antithrombotic	0.80	0.31–2.19	0.662	0.83	0.25–2.91	0.758
Antiplatelet	0.49	0.17–1.29	0.149	0.43	0.11–1.42	0.168
Anticoagulant	2.79	0.72–9.11	0.129	3.38	0.69–12.9	0.121

**Abbreviations:** 95% CI, 95% confidence interval; OR, odds ratio.

## Discussion

Interruption of antithrombotic therapy in the perioperative period has been reported to increase the risk of thromboembolism. 7 Additionally, according to the guidelines of the American College of Cardiology (ACC)/American Heart Association (AHA), the risk of cardiac complications in head and neck surgery is moderate (1–5%), 8 and the increased risk of thromboembolism due to discontinuation of antithrombotic agents must be carefully considered. However, postoperative bleeding in head and neck surgery is a serious complication that requires urgent intervention because it may lead to fatal adverse events, so the bleeding risk should also be carefully evaluated. For benign tumors, avoiding surgery or delaying surgery until antithrombotic therapy can be discontinued is an option; for malignant tumors, avoiding or delaying surgery may affect patient survival. Therefore, it is desirable to minimize the impact on treatment decisions.

The BRIDGE trial, 9 published in 2015, reported that perioperative continuation of VKA in patients with atrial fibrillation was noninferior to discontinuation of VKA plus heparin bridging for the prevention of perioperative thromboembolism and significantly reduced the rate of major bleeding. Additionally, a prospective observational study in Japan 10 found that in patients receiving antithrombotic therapy, discontinuation of antithrombotic agents prior to invasive procedures, including surgery, resulted in the development of thromboembolism, hemorrhagic complications, and death compared with continuation of antithrombotic therapy. However, this result was biased because it was left to physicians to decide whether to continue or discontinue antithrombotic therapy on a case-by-case basis. In addition, the continuation group was limited to about 10% of patients undergoing invasive procedures lasting more than one hour. Since 2015, we have in principle performed surgery while continuing antithrombotic therapy, and in practice, approximately 95% of the patients receiving antithrombotic therapy underwent surgery while continuing antithrombotic therapy. Because a relatively large number of patients underwent surgery while continuing antithrombotic agents without being discriminated by physicians, our study seems to more directly reflect the effects of antithrombotic agents.

In the present study, there was no difference in the incidence of postoperative bleeding between the continuation group and the discontinuation group for either antiplatelet or anticoagulant agents. Additionally, with regard to antiplatelet agents, no increase in postoperative bleeding was observed even after surgery when thienopyridines, which have a stronger antiplatelet effect, were continued.

In the observational study of patients receiving antithrombotic therapy, it was reported that the frequency of bleeding events was lower in the group receiving antiplatelet agents than in the one receiving VKA. 11 In areas other than head and neck surgery, there are some reports that the continuation of antiplatelet therapy in the perioperative period does not increase the risk of bleeding. 12 13 It can be concluded that surgery while continuing antiplatelet agents of any type does not lead to an increased risk of bleeding complications.

With regard to anticoagulant agents, it was difficult to evaluate due to the small number of cases and the bias in continuation and discontinuation according to the type of anticoagulant agents, but the frequency of postoperative bleeding was higher in the continuation group than in the discontinuation group, although there was no significant difference.

Regarding the type of anticoagulant agents, VKA has been used for many years, but it has drawbacks such as interactions with other drugs and diet, individual differences in metabolism, and the need for monitoring due to a narrow effective therapeutic range. Since 2011, however, DOACs have emerged that act directly and inhibit specific coagulation factors. They do not have this interaction and have a rapid onset of action and a short half-life. Therefore, the anticoagulant effect disappears rapidly after oral administration is discontinued. 14 Because of these properties, it has become popular as an alternative to VKA.

In a study on the management of perioperative DOACs, 15 even in surgeries with a relatively high bleeding risk, such as head and neck surgery, both postoperative bleeding and thromboembolism were like or lower than those in the BRIDGE study mentioned above, by setting a drug interruption/resumption period according to the half-life of each DOAC. Further consideration is needed for the perioperative management of anticoagulants, including DOACs, in the future.

To date, there are no reports of perioperative management of head and neck surgery without arbitrary decisions to continue or discontinue antithrombotic therapy. We believe that our study demonstrates that antiplatelet agents, regardless of type, do not increase the incidence of bleeding complications with continued use in surgery.

The present study has some limitations. First, due to the nature of retrospective studies, there is inevitably a selection bias, including the fact that the discontinuation group to which the continuation group is compared is heavily biased toward the first half of the study period. Certainly, since the time periods in which the groups exist are significantly different, the influence of the development of surgical instruments such as sealing devices and differences in surgeons may well be present. However, it also appears that surgery, at least with currently available instruments, does not lead to an increase in bleeding complications when antiplatelet agents are continued in the perioperative period.

Second, there are various factors other than antithrombotic agents that influence bleeding complications, but very few studies have been done due to the small number of cases. We believe there is no difference in bleeding complications between continuing and discontinuing of antiplatelet agents, but we have not been able to demonstrate the impact of continuing anticoagulant agents in the perioperative period because the number of patients taking anticoagulant agents is particularly small.

Therefore, we believe it is necessary to design a randomized, multicenter, prospective study based on the present study and explore better perioperative management of antithrombotic agents, especially anticoagulant agents.

## Conclusion

Surgery for head and neck malignancies with continued antithrombotic therapy did not increase surgical complications, including postoperative bleeding. In particular, antiplatelet agents, regardless of type and even when multiple antiplatelet agents were used, did not increase the risk of postoperative bleeding with continued antiplatelet therapy.
